# Selecting bioactive phenolic compounds as potential agents to inhibit proliferation and VEGF expression in human ovarian cancer cells

**DOI:** 10.3892/ol.2014.2818

**Published:** 2014-12-22

**Authors:** ZHIPING HE, BO LI, GARY O. RANKIN, YON ROJANASAKUL, YI CHARLIE CHEN

**Affiliations:** 1 Key Laboratory for Quality Improvement of Agricultural Products of Zhejiang Province, College of Agriculure and Food Science, Zhejiang A & F University, Lin’an, Zhejiang 311300, P.R. China; 2 College of Science, Technology and Mathematics, Alderson Broaddus University, Philippi, WV 26416, USA; 3 Department of Pharmacology, Physiology and Toxicology, Joan C. Edwards School of Medicine, Marshall University, Huntington, WV 25701, USA; 4 Department of Basic Pharmaceutical Science, West Virginia University, Morgantown, WV 26506, USA

**Keywords:** phenolic compound, ovarian cancer, cell viability, vascular endothelial growth factor, hypoxia-inducible factor-1α

## Abstract

Ovarian cancer is a disease that continues to cause mortality in female individuals worldwide. Ovarian cancer is challenging to treat due to emerging resistance to chemotherapy, therefore, the identification of effective novel chemotherapeutic agents is important. Polyphenols have demonstrated potential in reducing the risk of developing numerous types of cancer, as well reducing the risk of cancer progression, due to their ability to reduce cell viability and vascular endothelial growth factor (VEGF) expression. In the present study, eight phenolic compounds were screened in two human ovarian cancer cell lines (OVCAR-3 and A2780/CP70) to determine their effect on proliferation suppression and VEGF protein secretion inhibition, in comparison to cisplatin, a conventional chemotherapeutic agent. The current study identified that 40 μM gallic acid (GA) exhibited the greatest inhibitory effect on OVCAR-3 cell viability, compared with all of the phenolic compounds investigated. Similarly to cisplatin, baicalein, GA, nobiletin, tangeretin and baicalin were all identified to exhibit significant VEGF inhibitory effects from ELISA results. Furthermore, western blot analysis indicated that GA effectively decreased the level of the VEGF-binding protein hypoxia-inducible factor-1α in the ovarian cancer cell line. Considering the results of the present study, GA appears to inhibit cell proliferation and, thus, is a potential agent for the treatment of ovarian cancer.

## Introduction

Phenolic compounds, a large family of natural compounds with phenolic hydroxyl groups, are present in plants, fruits, vegetables and teas, and have been demonstrated to exhibit anticancer properties. The potential application of these phenolic compounds in the development of therapeutic agents for cancer treatment has gained increasing importance in the previous decade and research in this field is currently expanding (1–3). For example, Link *et al* (4) reported that cancer chemoprevention using dietary phenolic compounds may be important in the control of gene expression, for example for inducing changes in DNA methylation, histone modifications and non-coding RNAs. Furthermore, Mahbub *et al* (5) proposed that phenolic compounds may be potential therapeutic agents for the treatment of leukemia by causing decreased cell viability and inducing cell apoptosis. Additionally, previous studies have indicated that phenolic compounds are possibly associated with a reduced risk of liver (6), lung (7), cervical (8) and colorectal cancer (9).

Ovarian cancer is the second most lethal gynecologic cancer amongst the female population in developed areas (10), with an estimated 22,240 new cases and 14,030 mortalities due to ovarian cancer in 2013 (11). While the five-year survival rate of ovarian cancer patients has improved following the development of more effective treatment strategies with optimized therapeutic agent treatment and more advanced surgical techniques, the overall cure rate has remained ~30% over the previous two decades (12). An additional challenge for improving the five-year survival rate of ovarian cancer is that only 20% of ovarian cancers are diagnosed prior to the occurrence of metastasis (12), as the symptoms do not typically present until after the cancer has metastasized from the ovaries to the surface of the peritoneal cavity. Once metastasis has occurred, treatment of ovarian cancer is difficult as the surgical removal of all of the lesions is no longer possible and chemotherapy is the only remaining first-line treatment strategy.

Although many female individuals may respond well to initial first-line treatment, relapse frequently occurs with chemotherapy-resistant disease, presenting a major obstacle in the attempt to improve the prognosis of ovarian cancer patients (13). Therefore, selecting novel chemicals from natural compounds as cancer therapeutic agents remains important for ovarian cancer research. In particular, the discovery of novel natural compounds that meet or exceed the effects of commonly used chemical agents or agents that may be administered in combination with cisplatin to overcome the resistance, are of great significance (14). Currently, platinum agents, such as cisplatin, are one of the most active anticancer agents used in clinical practice (13).

Traditional plant-based medicines are widely used in developing countries for their primary healthcare requirement (15). The European Prospective Investigation cohort study into cancer and nutrition demonstrated that consuming >6.2 g per day of nuts and seeds appear to reduce a female individual’s risk of developing colorectal cancer (16). In analyses of pecan extract from various cultivars, (+)-catechin hydrate, ellagic acid, (−)-epicatechin and gallic acid (GA) were identified to be the main phenolic compounds (17,18). Furthermore, nobiletin, tangeretin, baicalein and baicalin were the predominant flavonoids identified in the common traditional Chinese medicines dried orange peel (citrus) and *Scutellaria*, respectively (19,20). These eight non-toxic dietary phenolic compounds have previously demonstrated anticancer and chemopreventive properties in specific types of cancer; however, little research has been conducted into the chemopreventive effects of these eight phenolic compounds against ovarian cancer.

**Figure 1:**
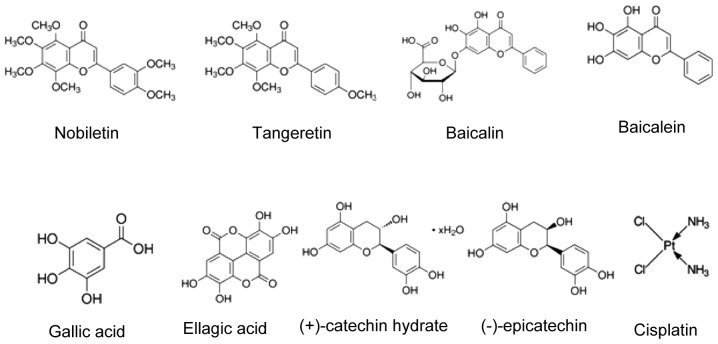
Chemical structures of the polyphenols and control compound (cisplatin) used to investigate cell viability and inhibition of vascular endothelial growth factor protein expression in ovarian cancer cell lines.

In the present study, the effect of eight dietary phenolic compounds on the cell proliferation and vascular endothelial growth factor (VEGF) protein expression levels in human ovarian cancer cells was investigated. The phenolic compounds investigated were (+)-catechin hydrate, ellagic acid, (−)-epicatechin, gallic acid, nobiletin, tangeretin, baicalein and baicalin, the names and structures of which are indicated in Fig. 1. Cisplatin, a commonly used chemotherapeutic agent, was used as the positive control.

## Materials and methods

### Materials and cell culture

The eight phenolic compounds and cisplatin were obtained from Sigma-Aldrich (St. Louis, MO, USA). The compounds were dissolved in dimethyl sulfoxide (DMSO) to produce stock solutions of 100 mM, and these stock solutions were subsequently diluted to 20 or 40 μM with cell culture medium prior to use. An equal amount of DMSO was included in the control solutions for every experiment. The two ovarian cancer cell lines OVCAR-3 and A2780/CP70 were provided by Dr Bing Hua Jiang at West Virginia University (Morgantown, WV, USA) and were maintained in RPMI-1640 medium (Sigma-Aldrich) supplemented with 10% fetal bovine serum (Invitrogen Life Technologies, Grand Island, NY, USA) in a cell culture incubator at 5% CO_2_ and a temperature of 37°C.

### Cell viability assay

To determine cell viability, the two cell lines were seeded in 96-well plates at a density of 1×10^4^ cells per well, and allowed to attach to the substrate and grown to log phase overnight. Following incubation at 37°C, the culture medium was removed and incubated with each of the nine compounds at a concentration of 20 or 40 μM in RPMI-1640 medium for 24 h. Each experiment was performed in triplicate. Following treatment, the cells were washed twice with phosphate-buffered saline (PBS; Invitrogen Life Technologies) and introduced with 100 μl freshly prepared CellTiter 96^®^ AQueous One solution (containing MTS, a tetrazolium compound; Promega Corporation, Madison, WI, USA) and 80 μl PBS. The cells were incubated for 1 h and the optical density values were measured at an absorbance of 490 nm using an ELISA plate reader (BioTek Instruments, Inc., Winooske, VT, USA). Cell viability was expressed as a percentage of the control.

### VEGF protein quantification

The effects of the nine compounds on VEGF protein secretion were analyzed by performing an ELISA with a Quantikine Human VEGF immunoassay kit (R&D Systems, Inc., Minneapolis, MN, USA), targeting VEGF165 in the cell culture supernatant. Cells (6×10^5^) from the two cell lines were seeded in 60-mm cell culture dishes and allowed to attach to the substrate and grow for 16 h prior to treatment with 40 μM of each compound or without, which served as a control, for an additional 24 h. The culture supernatants were collected for the VEGF assay and the inhibition of VEGF protein secretion was expressed as a percentage of the control.

### Western blot analysis

OVCAR-3 cancer cells were seeded and incubated overnight prior to treatment with 0, 5, 10 and 20 μM GA. The cells were double-washed with cold PBS, harvested with M-PER^®^ Mammalian Protein Extraction Reagent supplemented with Halt™ protease and phosphatase inhibitor cocktail, and the total protein expression levels were assayed using a bicinchoninic acid protein assay kit (Pierce Biotechnology, Inc., Rockford, IL, USA). The cell lysates (50 μg total protein) were separated by performing SDS-PAGE and blotted onto a nitrocellulose membrane using the Mini-PROTEAN 3 system (Bio-Rad Laboratories, Hercules, CA, USA). For immunodetection, mouse anti-human monoclonal HIF-1α (cat. no. 610959; dilution 1:500; BD Biosciences, Franklin Lakes, NJ, USA) and mouse anti-human monoclonal GAPDH antibodies (cat. no. sc-47724; dilution 1:500; Santa Cruz Biotechnology, Inc., Santa Cruz, CA, USA) were applied and the signals were visualized by phycoerythrin-conjugated goat anti-mouse IgG polyclonal secondary antibody (cat. no. 32230; dilution 1:2500; Pierce Biotechnology, Inc.) and Supersignal West Pico Chemiluminescent substrate application, followed by the utlilization of X-ray films (Pierce Biotechnology, Inc.). The protein bands were quantified using National Institutes of Health ImageJ software version 1.46 (Bethesda, MD, USA) and normalized by GAPDH bands for subsequent analysis.

### Statistical analysis

One-way and two-way analysis of variance (ANOVA) followed by Student-Newman-Keuls tests were applied to compare the effects of the nine compounds on cell viability and VEGF protein expression levels. All statistical analyses were performed using SAS software (SAS Institute, Inc., Cary, NC, USA). P<0.05 was considered to indicate a statistically significant difference.

## Results

### Comparison of the effects of various natural compounds on cell viability in OVCAR-3 ovarian cancer cells

**Figure 2:**
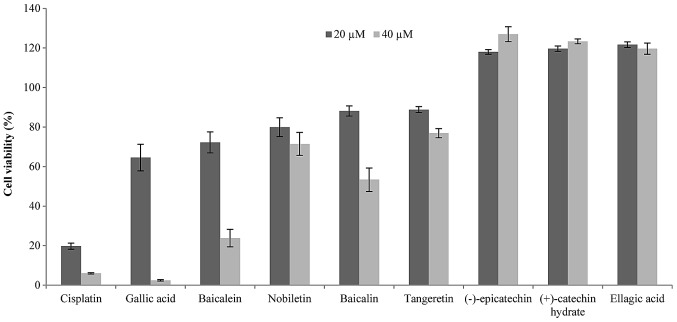
Effect of different compounds on cell viability in OVCAR-3 cells. Data are presented as the mean ± standard error of the mean from three independent experiments.

The 24-h treatment of OVCAR-3 cells with 20 and 40 μM of the nine different compounds resulted in various effects on OVCAR-3 cancer cell proliferation. As indicated in Fig. 2, (−)-epicatechin, (+)-catechin hydrate and ellagic acid caused no significant inhibition of OVCAR-3 cell proliferation; at 20 μM the cell viability ranged from 118.0±1.2 to 121.7±1.6% and at 40 μM from 119.7±2.8 to 127.0±3.8%. By contrast, GA was identified to exhibit the lowest cell viability value among all of the phenolic compounds, demonstrating an inhibitory effect similar to that of cisplatin at 40 μM. These results indicate that GA may have chemotherapeutic potential against ovarian cancer. Baicalin, baicalein, nobiletin and tangeretin exhibited a moderate inhibitory effect on cell proliferation between the levels of 72.2 and 88.8% at 20 μM and between 23.8 and 76.9% at 40 μM concentrations. According to the mean values of the two-way ANOVA results from the 20- and 40-μM treatments (Table I), the rank order of cell viability inhibition in OVCAR-3 cancer cells was as follows: Cisplatin > GA, baicalein > baicalin, nobiletin, tangeretin > ellagic acid, (+)-catechin hydrate and (−)-epicatechin (P<0.05).

### Comparison of the effects of natural compounds on cell viability in A2780/CP70 ovarian cancer cells

**Figure 3:**
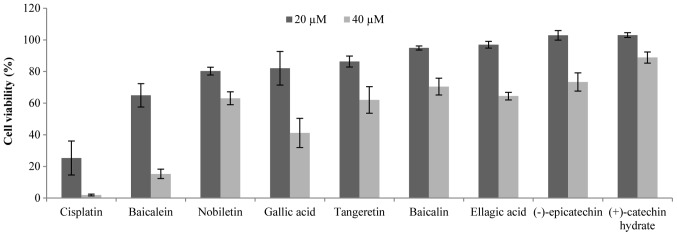
Effect of different compounds on A2780/CP70 cell viability. Data are presented as the mean ± standard error of the mean from three independent experiments.

The results of the cell viability assay indicated that the nine different compounds at the two stated concentrations exhibited varied effects on viability of ovarian cancer A2780/CP70 cells (Fig. 3). All of the phenolic compounds demonstrated inhibitory effects on the A2780/CP70 cells, with a cell viability range of 15.30±3.01 to 88.83±3.55% at 40 μM. This inhibitory mechanism of phenolic compounds may be due to their antioxidant activity, which would affect the cellular redox state of the cancer cells (21). All of the cell viability values of the investigated phenolic compounds were smaller than the values of cisplatin at 20 and 40 μM (P<0.05). Compared with the other phenolic compounds, GA and baicalein exhibited the greatest inhibitory effects at 40 μM (P<0.05); however, ellagic acid, baicalin, (−)-epicatechin and (+)-catechin hydrate were identified to have a smaller inhibitory effect on cell viability at 20 and 40 μM. The mean values of the two-way ANOVA analysis results from the 20- and 40-μM treatments indicated that the rank order of A2780/CP70 cell viability inhibition was as follows: Cisplatin > baicalein > GA ≥ nobiletin, tangeretin ≥ ellagic acid, baicalin, (−)-epicatechin ≥ (+)-catechin hydrate (P<0.05) (Table II).

### Comparison of the effects of natural compound on VEGF protein expression levels in the two ovarian cancer cell lines

**Figure 4:**
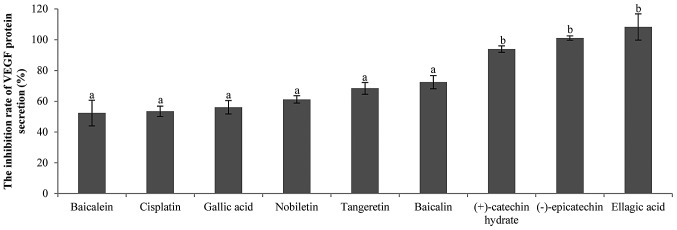
VEGF expression in OVCAR-3 cells treated with 40 μM of various compounds. Data are presented as the mean ± standard error of the mean from three independent experiments. Bars marked with different letters are significantly different (at P<0.05) when compared with each other.

**Figure 5:**
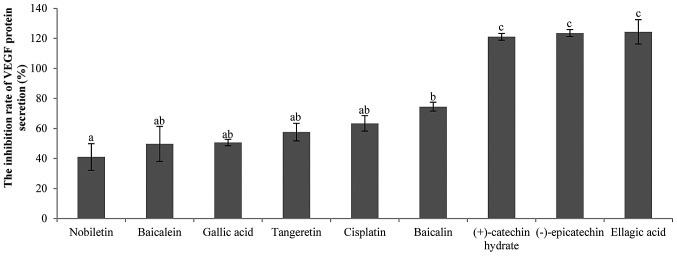
The effect of different compounds on VEGF expression in A2780/CP-70 cells at 40 μM. Data are presented as the mean ± standard error of the mean from three independent experiments. Bars marked with different letters are significantly different (at P<0.05) when compared with each other. VEGF, vascular endothelial growth factor.

The suppressive effects of each compound were measured by treating each cell line with 40 μM compounds for 24 h. In the OVCAR-3 cell line, baicalein, GA, nobiletin, tangeretin and baicalin all exhibited a moderate level of VEGF expression inhibition, similar to that of cisplatin, ranging from 52.4±14.5 to 72.4±7.3% (Fig. 4). As with the inhibitory effect on OVCAR-3 cancer cells (Fig. 5), GA, tangeretin, nobiletin, baicalein and baicalin were all identified to exhibit a moderate level of VEGF expression inhibition, ranging from 41.0±15.4 to 74.5±5.1%.

### Effect of GA on HIF-1α protein expression levels in OVCAR-3 cancer cells

GA, a type of phenolic acid, is present in a number of plant sources, such as the gall nut, sumac, witch hazel and various types of tea (22). Its anticancer activity has previously been reported in human glioma cells (22), as well as prostate (23), lung (24), gastric, colon, breast, cervical and esophageal cancer (25,26); however, the specific effect of GA on human ovarian cancer cells has not previously been reported. The present study demonstrated that GA markedly inhibits the proliferation of OVCAR-3 cells and significantly suppresses VEGF protein expression (P<0.05).

**Figure 6:**
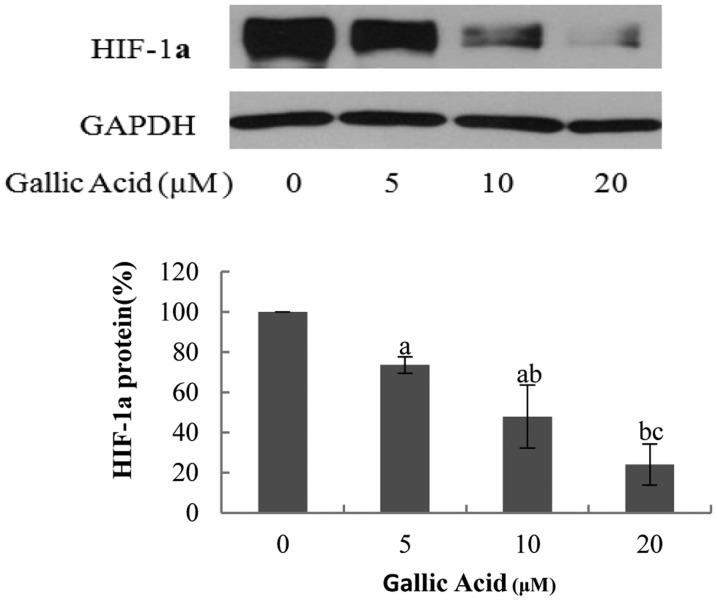
Gallic acid administration decreases HIF-1α protein expression levels in OVCAR-3 cells. Data represents the mean ± standard error of the mean from three independent experiments. Bars marked with different letters are significantly different (at P<0.05) when compared with each other. HIF-1α, hypoxia-inducible factor-1α.

The transcription factor HIF-1α has previously been reported to directly activate VEGF expression (27); therefore, the present study investigated the effect of GA on HIF-1α expression. In the OVCAR-3 cancer cell line, the protein expression levels of HIF-1α were significantly decreased (P<0.05) upon the administration of higher concentrations of GA (Fig. 6). At 20 μM GA, the protein expression levels of HIF-1α were 24.07% of the control, indicating that GA may effectively decrease the protein expression levels of HIF-1α in OVCAR-3 cells.

## Discussion

The results of the present study demonstrated that many of the compounds screened exhibit inhibitory effects on OVCAR-3 and A2780/CP70 ovarian cancer cell lines. It was identified that GA had the greatest inhibitory effect on OVCAR-3 cell viability, compared with all of the phenolic compounds investigated, with a similar viability to that of cisplatin at concentrations of 40 μM. Furthermore, the bioavailability of GA is high compared with the other polyphenols investigated (28), thus, GA is a potential agent for the prevention and treatment of human ovarian cancer. To the best of our knowledge, this is the first report to be conducted investigating the inhibitory effects of GA on ovarian cancer cells.

In the present study, all of the phenolic compounds investigated demonstrated similar levels of proliferative inhibition in the OVCAR-3 and A2780/CP70 cell lines, excluding GA. The cell viability of A2780/CP70 cells treated with 40 μM GA was markedly greater compared with the OVCAR-3 cells (P=0.014), which may indicate that the A2780/CP70 cells have reduced functional DNA mismatch repair, resulting in a decreased inhibitory effect of GA on ovarian cancer cells (29).

Baicalein, nobiletin, tangeretin, baicalin, (−)-epicatechin and (+)-catechin hydrate belong to a group of compounds called flavonoids, a subclass of polyphenols. These polyphenols all have a C6-C3-C6 structure with two benzene rings (A and B rings). The results of the present study indicated that the inhibitory effect of flavones (baicalein, nobiletin, tangeretin and baicalin) on OVCAR-3 and A2780/CP70 cancer cell lines was greater than that of flavanonols [(−)-epicatechin, (+)-catechin hydrate]. Of the flavones investigated, baicalein exhibited the greatest inhibitory effect, possibly due to baicalein containing a greater number of hydroxyls on the A ring compared with other flavones.

Typically, the majority of tumors grow to 1–2 mm, as they lack blood vessels which provide oxygen and the essential nutrients that are required for growth (30). Angiogenesis is a key process required for the delivery of nutrients and oxygen to the tumor nodule to allow the transition of a tumor from the dormant to malignant state. VEGF is a common growth factor that induces blood vessel growth in numerous types of cancer (31). Therefore, the identification of compounds capable of inhibiting VEGF secretion would be useful in preventing cancer growth. GA, tangeretin, nobiletin, baicalein and baicalin were identified to exhibit a moderate level of inhibition on the protein expression levels of VEGF, compared with the VEGF expression levels following ellagic acid, (+)-catechin hydrate and (−)-epicatechin treatment. These results may indicate that the inhibitory effect of flavones on OVCAR-3 and A2780/CP70 cancer cell lines is greater than the inhibitory effect of flavanonols.

Subsequent investigation identified that the protein expression levels of HIF-1α were dramatically decreased in OVCAR-3 cells from 100% (control) to 24.07% at 20 μM GA. OVCAR-3 cells were used as opposed to A2780/CP70 cells, as they exhibited lower resistance to GA. The results indicated that GA inhibits VEGF expression via the down-regulation of HIF-1α expression. In agreement with this hypothesis, previous studies have indicated that the inhibition of HIF-1α may be important in cancer therapy (32,33).

In conclusion, the results of this study indicate that GA exhibits an anti-angiogenic effect on ovarian cancer cells and thus, may be applied for use in human ovarian cancer prevention and therapy. However, future studies are required to explore the molecular mechanisms underlying GA’s anti-angiogenic effects.

## Figures and Tables

**Table I:** Effect of different compounds on OVCAR-3 cell viability.

	Cell viability, %		
	
Compound name	20 μM OVCAR-3	40 μM OVCAR-3	Mean
(−)-epicatechin	118.00±1.15^a^	127.01±3.79^a^	122.50^a^
(+)-catechin hydrate	119.67±1.33^a^	123.33±1.20^a^	121.50^a^
Ellagic acid	121.67±1.45^a^	119.67±2.85^a^	120.67^a^
Tangeretin	88.80±1.54^b^	76.90±2.29^b^	82.85^b^
Nobiletin	79.93±4.75^b,c^	71.47±5.81^b^	75.70^b^
Baicalin	88.13±2.56^b^	53.37±5.98^c^	70.75^b^
Baicalein	72.20±5.34^c,d^	23.80±4.40^d^	48.00^c^
Gallic acid	64.53±6.72^d^	2.43±0.34^e^	33.48^c^
Cisplatin	19.77±1.52^e^	6.02±0.37^e^	12.89^d^

Data are presented as the mean ± standard error of the mean from three independent experiment. Values marked with different letters are significantly different (at P<0.05) when compared with each other.

**Table II:** Effect of different compounds on A2780/CP70 cell viability.

	Cell viability, %		
	
Compound name	20 μM A2780/CP70	40 μM A2780/CP70	Mean
(+)-catechin hydrate	103.0±1.53^a^	88.83±3.55^a^	95.92^a^
(−)-epicatechin	102.8±3.03^a^	73.37±5.76^b^	88.1^a,b^
Baicalin	94.87±1.25^a^	70.43±5.31^b^	82.65^a,b^
Ellagic acid	96.90±2.11^a^	64.43±2.38^b^	80.67^a,b^
Tangeretin	86.33±3.46^a,b^	62.07±8.38^b^	74.2^b,c^
Nobiletin	80.27±2.48^a,b^	63.08±4.10^b^	71.68^b,c^
Gallic acid	82.03±10.66^a,b^	41.20±9.24^c^	61.62^c^
Baicalein	64.97±7.40^b^	15.30±3.01^d^	40.13^d^
Cisplatin	25.34±10.76^c^	1.92±0.54^d^	13.63^e^

Data are presented as the mean ± standard error of the mean from three independent experiment. Values marked with different letters are significantly different (at P<0.05) when compared with each other.

## References

[1] Henning SM, Wang P, Carpenter CL, Heber D (2013). Epigenetic effects of green tea polyphenols in cancer. Epigenomics.

[2] Wang ZJ, Ohnaka K, Morita M (2013). Dietary polyphenols and colorectal cancer risk: the Fukuoka colorectal cancer study. World J Gastroenterol.

[3] Abbas A, Patterson W, Georgel PT (2013). The epigenetic potentials of dietary polyphenols in prostate cancer management. Biochem Cell Biol.

[4] Link A, Balaguer F, Goel A (2012). Cancer chemoprevention by dietary polyphenols: promising role for epigenetics. Biochem Pharmacol.

[5] Mahbub AA, Le Maitre CL, Haywood-Small SL, McDougall GJ, Cross NA, Jordan-Mahy N (2013). Differential effects of polyphenols on proliferation and apoptosis in human myeloid and lymphoid leukemia cell lines. Anticancer Agents Med Chem.

[6] Stagos D, Amoutzias GD, Matakos A, Spyrou A, Tsatsakis AM, Kouretas D (2012). Chemoprevention of liver cancer by plant polyphenols. Food Chem Toxicol.

[7] Lee H, Kang C, Jung E, Kim JS, Kim E (2012). Antimetastatic activity of polyphenol-rich extract of Ecklonia cava through the inhibition of the Akt pathway in A549 human lung cancer cells. Food Chem.

[8] Di Domenico F, Foppoli C, Coccia R, Perluigi M (2012). Antioxidants in cervical cancer: chemopreventive and chemotherapeutic effects of polyphenols. Biochim Biophys Acta.

[9] Araújo JR, Gonçalves P, Martel F (2011). Chemopreventive effect of dietary polyphenols in colorectal cancer cell lines. Nutr Res.

[10] Jemal A, Bray F, Center MM, Ferlay J, Ward E, Forman D (2011). Global cancer statistics. CA Cancer J Clin.

[11] Siegel R, Naishadham D, Jemal A (2013). Cancer statistics, 2013. CA Cancer J Clin.

[12] Bast RC, Hennessy B, Mills GB (2009). The biology of ovarian cancer: new opportunities for translation. Nat Rev Cancer.

[13] Kigawa J (2013). New strategy for overcoming resistance to chemotherapy of ovarian cancer. Yonago Acta Med.

[14] Agarwal R, Kaye SB (2003). Ovarian cancer: strategies for overcoming resistance to chemotherapy. Nat Rev Cancer.

[15] Akter R, Uddin SJ, Grice ID, Tiralongo E (2014). Cytotoxic activity screening of Bangladeshi medicinal plant extracts. J Nat Med.

[16] Jenab M, Ferrari P, Slimani N (2004). Association of nut and seedintake with colorectal cancer risk in the European Prospective Investigation into Cancer and Nutrition. Cancer Epidemiol Biomarkers Prev.

[17] Villarreal-Lozoya JE, Lombardini L, Cisneros-Zevallos L (2007). Phytochemical constituents and antioxidant capacity of different pecan [Carya illinoinensis (Wangenh.) K Koch] cultivars. Food Chem.

[18] Wu X, Beecher GR, Holden JM, Haytowitz DB, Gebhardt SE, Prior RL (2004). Lipophilic and hydrophilic antioxidant capacities of common foods in the United States. J Agr Food Chem.

[19] Mencherini T, Campone L, Piccinelli AL, Mesa MG, Sánchez DM, Aquino RP, Rastrelli L (2013). HPLC-PDA-MS and NMR characterization of a hydroalcoholic extract of Citrus aurantium L. var. amara peel with antiedematogenic activity. J Agric Food Chem.

[20] Yu MW, Lou SN, Chiu E, Ho CT (2013). Antioxidant activity and effective compounds of immature calamondin peel. Food Chem.

[21] Mitjavila MT, Moreno JJ (2012). The effects of polyphenols on oxidative stress and the arachidonic acid cascade. Implications for the prevention/treatment of high prevalence diseases. Biochem Pharmacol.

[22] Lu Y, Jiang F, Jiang H, Wu K, Zheng X (2010). Gallic acid suppresses cell viability, proliferation, invasion and angiogenesis in human glioma cells. Eur J Pharmacol.

[23] Kaur M, Velmurugan B, Rajamanickam S, Agarwal R, Agarwal C (2009). Gallic acid, an active constituent of grape seed extract, exhibits anti-proliferative, pro-apoptotic and anti-tumorigenic effects against prostate carcinoma xenograft growth in nude mice. Pharm Res.

[24] You BR, Kim SZ, Kim SH, Park WH (2011). Gallic acid-induced lung cancer cell death is accompanied by ROS increase and glutathione depletion. Mol Cell Biochem.

[25] You BR, Moon HJ, Han YH, Park WH (2010). Gallic acid inhibits the growth of HeLa cervical cancer cells via apoptosis and/or necrosis. Food Chem Toxicol.

[26] Faried A, Kurnia D, Faried LS, Usman N, Miyazaki T, Kato H, Kuwano H (2007). Anticancer effects of gallic acid isolated from Indonesian herbal medicine, Phaleria macrocarpa (Scheff.) Boerl, on human cancer cell lines. Int J Oncol.

[27] Luo H, Li B, Li Z, Cutler SJ, Rankin GO, Chen YC (2013). Chaetoglobosin K inhibits tumor angiogenesis through downregulation of vascular epithelial growth factor-binding hypoxia-inducible factor 1α. Anticancer Drugs.

[28] Roberts AT, Martin CK, Liu Z, Amen RJ (2007). The safety and efficacy of a dietary herbal supplement and gallic acid for weight loss. J Med Food.

[29] Strathdee G, MacKean MJ, Illand M, Brown R (1999). A role for methylation of the hMLH1 promoter in loss of hMLH1 expression and drug resistance in ovarian cancer. Oncogene.

[30] Folkman J (1990). What is the evidence that tumors are angiogenesis dependent?. J Natl Cancer Inst.

[31] Weng CJ, Yen GC (2012). Chemopreventive effects of dietary phytochemicals against cancer invasion and metastasis: phenolic acids, monophenol, polyphenol, and their derivatives. Cancer Treat Rev.

[32] Semenza GL (2003). Targeting HIF-1 for cancer therapy. Nat Rev Cancer.

[33] Semenza GL (2009). HIF-1 Inhibitors for cancer therapy: from gene expression to drug discovery. Curr Pharm Des.

